# Quantifying sources of bias in longitudinal data linkage studies of child abuse and neglect: measuring impact of outcome specification, linkage error, and partial cohort follow-up

**DOI:** 10.1186/s40621-017-0119-6

**Published:** 2017-08-07

**Authors:** Jared W. Parrish, Meghan E. Shanahan, Patricia G. Schnitzer, Paul Lanier, Julie L. Daniels, Stephen W. Marshall

**Affiliations:** 1 Alaska Division of Public Health, Section of Women’s, Children’s, and Family Health, 3601 C St., Suite 358, Anchorage, AK 99503 USA; 2 The University of North Carolina at Chapel Hill, School of Public Health, 2101 McGavran-Greenberg Hall, CB# 7435, Chapel Hill, NC 27599 USA; 3 The National Center for the Review & Prevention of Child Deaths, c/o Michigan Public Health Institute, 1115 Massachusetts Ave. NW, Washington, DC 20005 USA; 4 The University of North Carolina at Chapel Hill, School of Social Work, 325 Pittsboro St. #3550, Chapel Hill, NC 27516 USA; 5 The University of North Carolina at Chapel Hill, Injury Prevention Research Center, 137 East Franklin St, CB# 7505, Chapel Hill, NC 27599 USA

**Keywords:** Child maltreatment, Record linkage, Birth cohort, Longitudinal study, Bias, Health informatics, PRAMS

## Abstract

**Background:**

Health informatics projects combining statewide birth populations with child welfare records have emerged as a valuable approach to conducting longitudinal research of child maltreatment. The potential bias resulting from linkage misspecification, partial cohort follow-up, and outcome misclassification in these studies has been largely unexplored. This study integrated epidemiological survey and novel administrative data sources to establish the Alaska Longitudinal Child Abuse and Neglect Linkage (ALCANLink) project. Using these data we evaluated and quantified the impact of non-linkage misspecification and single source maltreatment ascertainment use on reported maltreatment risk and effect estimates.

**Methods:**

The ALCANLink project integrates the 2009–2011 Alaska Pregnancy Risk Assessment Monitoring System (PRAMS) sample with multiple administrative databases through 2014, including one novel administrative source to track out-of-state emigration. For this project we limited our analysis to the 2009 PRAMS sample. We report on the impact of linkage quality, cohort follow-up, and multisource outcome ascertainment on the incidence proportion of reported maltreatment before age 6 and hazard ratios of selected characteristics that are often available in birth cohort linkage studies of maltreatment.

**Results:**

Failure to account for out-of-state emigration biased the incidence proportion by 12% (from 28.3%_w_ to 25.2%_w_), and the hazard ratio (HR) by as much as 33% for some risk factors. Overly restrictive linkage parameters biased the incidence proportion downwards by 43% and the HR by as much as 27% for some factors. Multi-source linkages, on the other hand, were of little benefit for improving reported maltreatment ascertainment.

**Conclusion:**

Using the ALCANLink data which included a novel administrative data source, we were able to observe and quantify bias to both the incidence proportion and HR in a birth cohort linkage study of reported child maltreatment. Failure to account for out-of-state emigration and low-quality linkage methods may induce bias in longitudinal data linkage studies of child maltreatment which other researchers should be aware of. In this study multi-agency linkage did not lead to substantial increased detection of reported maltreatment. The ALCANLink methodology may be a practical approach for other states interested in developing longitudinal birth cohort linkage studies of maltreatment that requires limited resources to implement, provides comprehensive data elements, and can facilitate comparability between studies.

**Electronic supplementary material:**

The online version of this article (doi:10.1186/s40621-017-0119-6) contains supplementary material, which is available to authorized users.

## Background

Child maltreatment, which includes all forms of abuse, neglect, and mental injury of a child by a parent or other caregiver, is under-studied relative to its public health significance, impact on children, and contribution to adult health outcomes (Butchart et al. 2006; Leeb et al. 2008). Given the complex etiologies contributing to maltreatment, it is important to focus and evaluate prevention efforts using analytic models that utilize population representative longitudinal data sources (Cicchetti and Carlson 1989; Cicchetti 1994). Current nationally available data on maltreatment such as those collected and reported by the National Child Abuse and Neglect Data System (NCANDS) and National Incidence Study (NIS) do not allow for longitudinal assessment but provide annual snapshots (US Department of Health and Human Services et al. 2016; Sedlak et al. 2010a). Studying maltreatment, especially at the population level and over time is challenging but necessary to quantify risk (Rothman et al. 2008). Due to the many conceptual and logistical challenges of conducting population level longitudinal maltreatment research, traditional prospective cohort studies are often limited to subset populations known to child welfare (e.g. The Longitudinal Studies on Child Abuse and Neglect) (Runyan et al. 1998; Bertolli et al. 1995; Brownell and Jutte 2013). Large population representative longitudinal cohort studies are expensive, time-consuming, and require extensive administrative support to conduct participant follow-up (Rothman et al. 2008). Due to these and other challenges, alternative methods for generating population-representative longitudinal studies to examine child maltreatment are necessary.

Accordingly, linkage projects combining statewide birth records with child protective services (CPS) records have emerged as a health informatics approach (Putnam-Hornstein et al. 2013a; Jonson-Reid and Drake 2008; Wu et al. 2004; Jutte et al. 2011; Stanley et al. 1994). Birth cohort linkages in Australia, New Zealand and Canada have demonstrated the benefit of studying maltreatment through administrative record linkages (Brownell and Jutte 2013; Holman et al. 1999; Tonmyr et al. 2014). In the US, linkage studies of full birth cohorts in California, Florida, Texas, and Alaska have highlighted the promise of this approach to study many child health outcomes and measure the incidence proportion of maltreatment over time (Wu et al. 2004; Van Horne et al. 2015; Putnam-Hornstein and Needell 2011; Gessner et al. 2004).

The use of entire statewide birth cohorts typically results in good statistical precision; these results however, are still subject to systematic error (Bertolli et al. 1995). Bias may result from a number of factors (Bohensky 2016), including 1) the influence of unknown selection factors with registration on administrative databases (e.g. institutional racism that can lead to biased reporting, or regional variation in applying screening policies), 2) pragmatic difficulties associated with accurately tracking all subjects over time using routine administrative databases (e.g. no access to or source of annual or regularly updated population level administrative data) resulting in unmeasured loss-to-follow up, 3) incomplete covariate adjustment for predictive and etiologic assessments due to limitations in availability and scope of data elements (e.g. birth certificates provide limited prenatal, social, and behavioral information and can limit etiologic and predictive modeling), 4) reliance on official reports of maltreatment to capture the outcome (Official reports to child welfare agencies are known to under-represent the magnitude of the problem due to under-reporting) (Sedlak et al. 2010b; Ewigman et al. 1993; Drake and Zuravin 1998), and 5) linkage misspecifications (e.g. using restrictive linkage assumptions when integrating data or having limited capacity for manual review of partial matches, no access to name change records, and large subpopulations with differential linkage patterns due to name homogeneity). Limited research to date has assessed the influence of these sources of bias on population based child maltreatment data linkage studies (Bohensky 2016; Greene et al. 2011).

We recently piloted a novel data linkage approach based on the methodology suggested by Bertolli et al. nearly 2 decades ago for studying child maltreatment (Bertolli et al. 1995). Our pilot project integrated the Pregnancy Risk Assessment Monitoring System (PRAMS) sample in Alaska with CPS reports occurring after birth (Parrish et al. 2011) and demonstrated comparable associations to those published in the literature using full birth cohorts (Wu et al. 2004; Putnam-Hornstein and Needell 2011).

This paper expands greatly upon the initial pilot studies and describes the creation of the Alaska Longitudinal Child Abuse and Neglect Linkage (ALCANLink) project that integrates epidemiologic survey and multi-sector administrative data (Calderwood and Lessof 2009) to create a comprehensive longitudinal birth cohort study. We highlight the benefit of the ALCANLink methodology by documenting the bias in incidence and hazard ratios that can arise in birth cohort linkage studies due to incomplete data linkages, nonlinkage assumptions, and single source outcome ascertainment.

## Methods

**Fig. 1:**
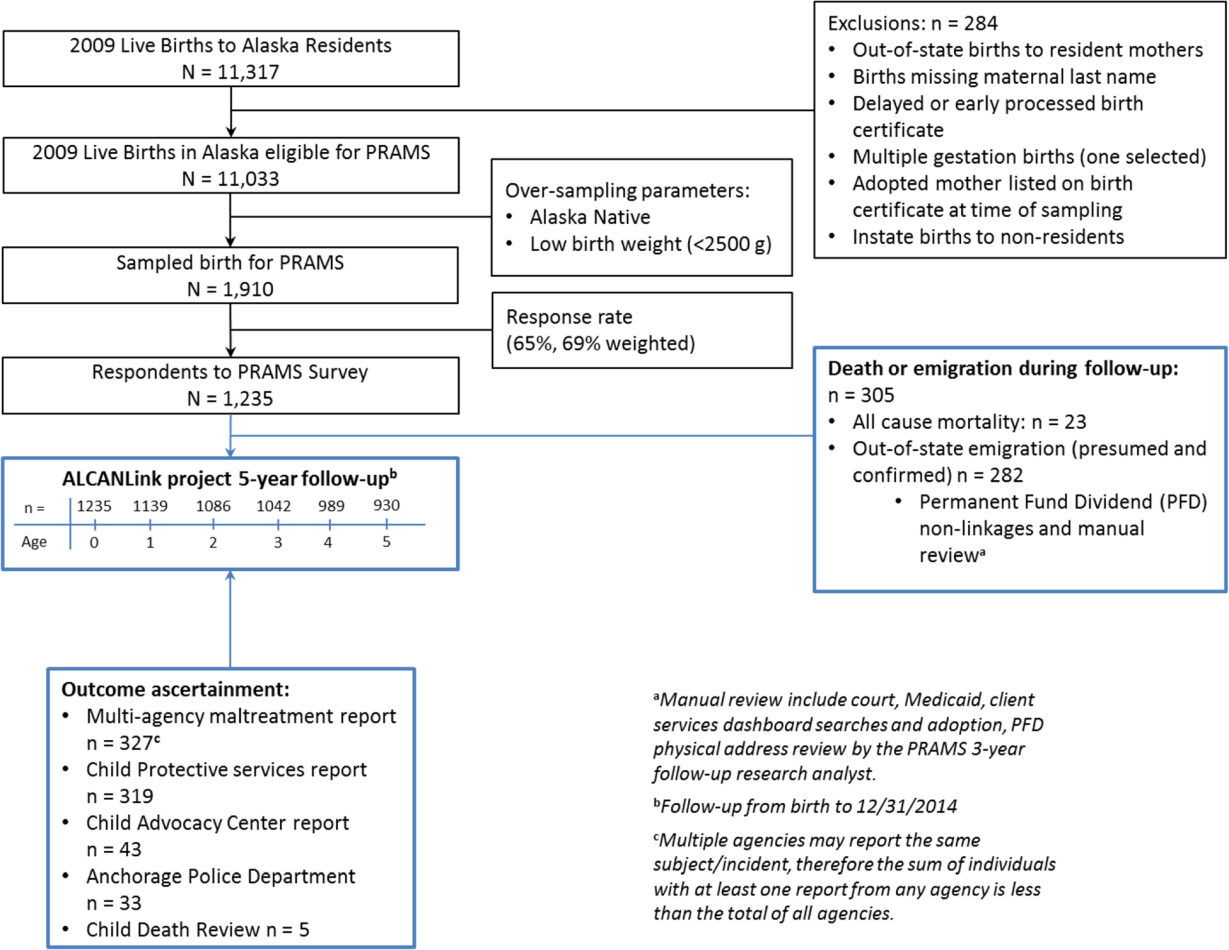
ALCANLink Project Participant Flow Diagram

The ALCANLink project integrates the 2009–2011 PRAMS respondent births (hereafter referred to as PRAMS births) with a core set of sources to follow the PRAMS cohort prospectively, which include vital records, child death review, and Alaska Permanent Fund Dividend (PFD) records (Alaska Department of Revenue: Permanent Fund Dividend Division 2016). Additional administrative sources and a three-year follow up survey to PRAMS capture additional factors. We limited our in-depth assessment described in this paper to a single PRAMS year (2009 births) to minimize the manual review and classification processes required, and allow for easier presentation of cohort details (Fig. 1).

### Cohort establishment

Alaska PRAMS uses a representative stratified systematic sample of annual resident live births. It oversamples Alaska Native mothers and low birth weight (<2500 g) infants for reasons of statistical precision. Oversampling and nonresponse are reflected in post-stratification sample weights. Alaska PRAMS samples approximately 1 in every 6 live births occurring to resident mothers, and maintains about a 65% weighted response rate. Complete PRAMS methodology is described in detail elsewhere (Shulman et al. 2006).

In 2009, 11,317 live births occurred among Alaska resident mothers, with 11,033 meeting PRAMS inclusion criteria. Alaska PRAMS attempted to survey 1910 (17.3%) of these eligible mothers of newborns, with 1235 (64.7%) responding to the survey (69% weighted response rate).

We used the PRAMS cohort as the basis of ALCANLink opposed to the full birth cohort for the following reasons: a) PRAMS provides population-representative exposure measures that are extensively more comprehensive and detailed than those available only on birth records, b) PRAMS respondents provide consent to have their responses linked to other information the department has about them facilitating data linkages with multiple administrative databases, c) PRAMS is conducted in nearly all other states potentially allowing for standardization and expansion of these methods, and d) The complex sampling enables population estimation while reducing the resources required for exhaustive data linkages which can ultimately make additional administrative data linkages unfeasible.

### Cohort follow-up

The 2009 PRAMS respondent births (*n* = 1235) were followed prospectively by linking PRAMS birth children to death certificates, Alaska Child Death Review (CDR) program records, and the Alaska Permanent Fund Dividend database with the most recent complete and available year (2014) at the time of this analysis. We used these follow-up sources to censor subjects for competing cause mortality and out-of-state emigration from Alaska.

In order to identify maltreatment-related child fatalities, we cross-checked all identified fatalities with the Alaska CDR program (see outcome ascertainment section below). All PRAMS births were subsequently linked to the annual PFD database. Adopted by constitutional amendment in 1976, Alaska established the Permanent Fund to invest a portion of the revenue earnings generated from petroleum production (Goldsmith 2002). The dividend is available, upon application, to all legal Alaskan residents with strict eligibility requirements. Infants born on or before December 31st of a qualifying year are eligible for a PFD. Since 2009, an average of 92.2% of the state population has applied for, and 86.0% approved for a dividend annually (Alaska Department of Revenue: Permanent Fund Dividend Division 2016). The PFD essentially serves as an annual census and therefore provides a unique source for conducting historical cohort studies using the Alaska population. We know of no other comparable epidemiologic resource for all residents within any other US state, which enables us to explicitly quantify the potential bias associated with linkage misspecification in longitudinal birth cohort linkage studies. PRAMS respondent children that failed to link with either death records or PFD records underwent an extensive manual review using multiple administrative state sources which included, child and parental searches in PFD, court, Medicaid and WIC records, and a state based master client index.

### Censoring & Competing Causes

PRAMS births censored due to competing causes of death (deaths not classified as maltreatment-related by the Alaska CDR committee) were followed and censored at the date of death. To detect out-of-state emigration, the PFD data was used. This source only allows for annual interval censoring, which required us to develop a set of rules for systematic classification (Table 1). For a visual depiction of these censoring rules please see Additional file 1: Figure S1.

**Table 1:** Censoring rules for ALCANLink cohort

Linkage observation	Censoring rule^a^
Linkage with vital death records and **not** classified by Alaska CDR as maltreatment related.	Censor at date of death
PRAMS births failing to link with any PFD year, or only linking with PFD records with only a non-Alaska address	Censor at 3-months from the date of birth
Last PFD indicates out-of-state residence or non-linkage with subsequent PFDs	Censor at the mid-year of the subsequent calendar year.
Linkage with last available PFD (2015 PFD year, 2014 calendar year)	Administrative censor at 12/31/2014

^a^For these rules we assumed a uniform distribution of out-of-state emigration during a calendar year. We also reasoned that (for example) as a 2011 PFD year application reflects residence during the 2010 calendar year, to be eligible for the 2010 PFD the subject must have physically resided in Alaska (aside for a few exceptions) the majority of that year and lost eligibility sometime during the subsequent year (2011)

The PFD provides only a crude annual estimation of censorship and even with manual review we are unable to identify exact dates, we examined multiple different censoring rules and compared the: 1) person-time estimation, 2) number of outcomes (reports of maltreatment) excluded based on the rule specification (recognizing that such exclusions may reflect lack of precision in the PFD and CPS dates), and 3) impact on the incidence estimate, crudely approximated as the number of events divided by the total person-time at risk (data presented in Additional file 1: Table S1). Based on the evaluated rule sets, our original *a priori* definition captured all outcomes within our observation window and censored for out-of-state emigration using a conservative rule to maximize accrual of person-time and was thus utilized as a reasonable expectation. Finally, we scrutinized, using the manual review process described above, a subset of cases that responded to the three-year follow-up survey but had a survey date greater than the last PFD linkage date (*n* = 23). Among these 23 cases, 18 had either moved or died, 3 were either adoptions or had substantial name changes and missed as initial linkages, and 2 had no documented history with PFD but remained in the state. Based on our censoring rule for these 23 cases we estimated a contribution of 126 person-months compared to 120 person-months calculated when using the most probable departure dates identified through manual review.

### Outcome ascertainment

Identifying and classifying maltreatment is problematic because it requires capturing an event that often occurs out of sight (US Department of Health and Human Services et al. 2016; Putnam-Hornstein and Needell 2011; Socolar et al. 1995; Dubowitz et al. 2005; Runyan et al. 2005; Hussey et al. 2005). Official reports, survey, medical records review, and multi-source data linkages have all been used to detect and classify maltreatment (Fluke et al. 2008). Official reports to child welfare agencies are known to under-represent the magnitude of the problem due to under-reporting (Ewigman et al. 1993; Drake and Zuravin 1998; Sedlak et al. 2010b). The process of screening and confirming maltreatment (substantiation) is influenced by policy, adequacy of information, and other external processes (Drake 1996). Although substantiations or confirmations are important, public health research has begun to shift towards the use of all recorded maltreatment reports by CPS agencies regardless of determination (Kohl et al. 2009). Studies document that children confirmed for maltreatment by child welfare experience similar negative health outcomes as those that are recorded but unconfirmed as well as those that are only reported but not evaluated for maltreatment (Parrish et al. 2011; Runyan et al. 2005; Hussey et al. 2005; Drake 1996; Putnam-Hornstein 2011; Leiter et al. 1994).

For the ALCANLink project and based the public health definitions proposed by CDC for classifying maltreatment (Leeb et al. 2008), we attempted to improve upon sole reliance on CPS records by broadening the range of agencies contributing maltreatment reports. In Alaska, state statute mandates that specified professionals (e.g. medical provider, education instructor), must report suspected maltreatment to the state child welfare agency. We developed a combined multi-agency reported maltreatment outcome measure to account for suspected non-reported maltreatment to CPS. The multi-agency measurement includes child welfare records (including both screened in and screened out reports), 8 of the 10 active Child Advocacy Center (CAC) agencies reports, the Anchorage Police Department (APD) which covers nearly 50% of Alaska’s population, and the Alaska Maternal Child Death Review (MCDR) maltreatment committee determinations. The Alaska MCDR committee reviews all child deaths occurring in Alaska and for each death classifies if any form of omission or commission caused or contributed to the death. Due to know underestimation of death certificate classifications and to be consistent with our sensitive reported maltreatment definition we included all deaths that the committee indicated abuse, neglect, or negligence “yes” or “yes probably” caused or contributed to the death. The CDC definitions provide a framework for quantifying potential maltreatment from a public health perspective and allow for a more sensitive cross-jurisdictional qualification of incidents (Jack 2010). For a more detailed description of the reported maltreatment classification see Additional file 1: Table S2.

### Linkage methods

We implemented both deterministic and probabilistic methods to link PRAMS births with each dataset. Prior to all linkages we conducted systematic record set cleaning, including date, character, and case equalization, standardization of missing data and treatment of special characters, and removal of leading/trailing spaces. Using iterative linkages (deterministic followed by probabilistic) we reduced the amount of suspected matches requiring manual review. For probabilistic linkages we developed comparison patterns based on a Joarowinkler distance metric to account for typos, spelling errors, transpositions, and other edits or deletions between two strings or set of strings and dates. The probabilistic linkage approach automatically accepted matches when the first, last, and alias names, date of birth and sex were identical. Suspected matches that returned a probability match score between 0.85 and 0.99 were manually reviewed, while those below 0.85 were automatically rejected. For complete linkage details and methods on establishment of these thresholds for review please see Data linkages in the Additional file 1. The RecordLinkage package (Sariyar and Borg 2010) in the R environment (R Core Team 2014) was used for all data linkages.

### Statistical analysis

We calculated the incidence proportion (“cumulative risk”) of first multi-source report of maltreatment before age six years. We estimated the survivorship function S(t) using a weighted Aalen hazard-based estimation (Klein and Moeschberger 2005) and 95% confidence interval on the log survival scale (Link 1984). We calculated the weighted cumulative distribution function F(t) from the weighted survivorship function S(t) [F(t) = 1 – S(t)]. We used weighted F(t) to estimate the incidence proportion of a multi-source maltreatment report before age six in the birth population. Frequency counts are presented as actual participant responses and weighted proportions from the complex sampling design are noted as %_w_.

We created a dichotomous variable for censorship (yes or no) to assess the probability of censorship for a limited number of selected covariates obtained from both the birth certificate and PRAMS responses using logistic regression. The limited set of covariates selected for investigation to assess this potential bias included: as a proxy for military families if the birth was paid by Tricare (yes, no); sex of the child (male, female); years of maternal education completed at delivery of child (<12 year, 12 + years); marital status at birth (married; unmarried); any maternal alcohol use during pregnancy as indicated on the birth certificate or PRAMS (yes, no); any maternal smoking during pregnancy as indicated on the birth certificate or PRAMS (yes, no); maternal race (Asian/Pacific Islander, Black, Native, White); birth defect indicated on the birth certificate (yes, no); mother or child on Medicaid at birth (yes, no); fathers name listed on birth certificate (yes, no); maternal age at birth (continuous); multi-agency maltreatment report (yes, no); mother reported being divorced/separated 12 months before pregnancy (yes, no); mother reported moving 12 months before pregnancy (yes, no); mother reported losing a job 12 months before pregnancy (yes, no); mother reported partner/husband losing a job 12 months before pregnancy (yes, no). These covariates were selected due to either being previously documented in the literature to be associated with maltreatment and hypothesized to potentially have differential population movement (Wu et al. 2004; Putnam-Hornstein and Needell 2011; Rentz et al. 2006; Putnam-Hornstein et al. 2013b). We then calculated and compared the incidence proportion and hazard ratio with and without out-of-state emigration to measure the impact of systematic bias on these selected values. We followed this same methodology to estimate the impact on both incidence proportion and hazard ratios assuming only deterministic linkages and reliance on CPS reported cases only, and in combination. All analyses were conducted in R 3.1.0 (R Core Team 2014) using the survey package (Lumley 2012).

## Results

We successfully matched 1162 (94.1%) of the 1235 PRAMS births to at least one PFD record with an Alaska residence before the age of 6 years. Among the 73 non-matching births, 15 were deaths occurring during the first year of life. On average, deterministic linkages captured 93.7% of all correct matches with annual PFD data. The PRAMS sample consistently linked with between 9% and 10% of PFD, CPS, APD, and CAC records (see Additional file 1: Table S3 for linkage rate details for ALCANLink project).

### Outcome ascertainment

**Fig. 2:**
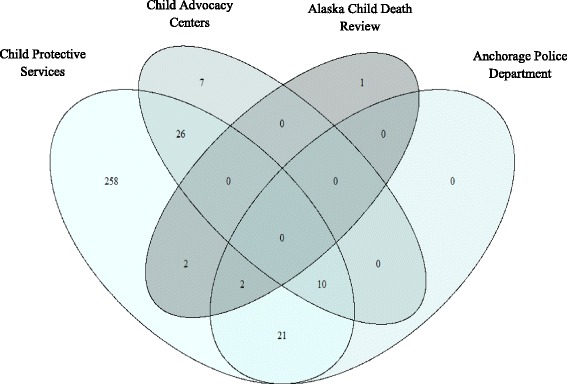
Detection of reported maltreatment by data source, ALCANLink 2009 (*n* = 327)

Among the 1235 PRAMS births, 327 (24.2%_w_) had at least one multi-source report of maltreatment during the follow-up period. Of the 327 multi-source reports detected, CPS captured the overwhelming majority (*n* = 319, 98%), CAC captured 43 (13%), APD captured 33 (10%), and the CDR captured five (2%) fatalities (Fig. 2). The preponderance of reports occurred prior to age 1 year (39.1%_w_), and monotonically decreased to 10.6% through age 5 years. Among the 1235 PRAMS births and considering only documented reports by CPS, 2.7%_w_ (95% CI: 1.7%_w_, 6.5%_w_) were reported for alleged sexual abuse, 5.1%_w_ (95% CI: 3.6%_w_, 6.5%_w_) were reported for alleged physical abuse, 9.1%_w_ (95% CI: 7.2%_w_, 11.0%_w_) were reported for alleged mental injury, and 21.0%_w_ (95% CI: 18.4%_w_, 23.6%_w_) were reported for alleged neglect among the birth population. The majority reported to CPS were due to neglect (88.7%_w_), followed by mental injury (38.5%_w_), physical abuse (21.4%_w_), and sexual abuse (11.6%_w_; totals sum to greater than 100% due to children being reported for multiple types of maltreatment).

### Cohort follow-up

The cohort was followed for 5812.7 (86.9%) of the 6690.9 total potential person-years. Among the 1235 PRAMS births, 930 (75.3%) had complete cohort follow-up through the first 5 years of life. Approximately 4% of the births were lost-to-follow up annually. Among the 305 births lost-to-follow up during the project period regardless of outcome, 32% were lost prior to age 1 year and 49% prior to age 3 years. There were 23 total deaths, with 78% occurring prior to age 1 year. Cohort follow-up details are available in Table 2.

**Table 2:** Cohort follow-up by age in years to event, death, or censor, ALCANLink 2009 (*n* = 1235)

	[0–1)		[1–2)		[2–3)		[3–4)		[4–5)		[5–6)	
	N	%_w_	N	%_w_	N	%_w_	N	%_w_	N	%_w_	N	%_w_
Follow-Up												
Subjects entering age interval outcome free	1235	(100)	1017	(84.7)	917	(75.1)	831	(68.5)	747	(61.8)	674	(56.5)
Person-years accrued in interval	1118.4	(92.6)	968.3	(94.5)	871.3	(95.7)	788.7	(94.9)	708.6	(95.5)	325.2	(49.2)
Censoring												
Competing deaths	16	(77.8)	0	0	0	0	1	(11.1)	1	(11.1)	0	(0)
Loss to follow-up	67	(26.5)	45	(25.3)	37	(14.0)	41	(17.9)	36	(13.4)	11	(2.9)
Outcome Ascertainment												
First multi-source report^a^	135	(39.1)	54	(18.7)	48	(15.5)	42	(12.8)	36	(10.6)	12	(3.4)
CPS only report	131	(38.2)	53	(18.9)	48	(16.0)	41	(13.1)	34	(10.3)	12	(3.5)

^a^multi-source report is the first report of maltreatment to either Child Protective Services, Child Advocacy Center, Anchorage Police Department, or the Maternal Child Death Review

A total of 162 (14.5%_w_) PRAMS births were paid by TRICARE (crude proxy for military births). Military paid births had substantially more out-of-state emigration before age six (73.2%_w_ vs 17.0%_w_, *p* < 0.001), to such an extent that military paid births accounted for 42.5%_w_ of all emigration movements. Among military paid births only 54.6%_w_ of total potential person time was captured, compared with 91.9%_w_ among non-military births before age six. The proportion of first reported multi-agency events was slightly lower among military paid births compared to non-military births (18.4%_w_ vs 25.2%_w_, *p* = 0.183).

Among the selected covariates assessed, the odds of out-of-state emigration censorship was higher among military paid births, married mothers at birth, maternal Black race (relative to White), birth or pregnancy not being covered by Medicaid as indicated on the birth certificate, and maternal self-reporting husband or partner losing a job or reporting moving to a new address during the 12 months before the child’s birth. The odds of out-of-state emigration censorship was lower among children of Alaska Native mothers (relative to White) (Table 3).

**Table 3:** Unadjusted Odds of censorship among the PRAMS respondents, ALCANLink (*n* = 1235)

	Unweighted OR (95% CI)	pvalue	Weighted OR (95% CI	pvalue
Birth Paid by Tricare (ref = no)	12.3 (8.5, 18.1)	<0.001	13.3 (8.4, 21.8)	<0.001
Male Sex (ref = Female)	1.2 (0.9, 1.5)	0.301	1.3 (0.9, 1.8)	0.087
Maternal education 12 + years (ref = <12 years)	1.3 (0.9, 1.9)	0.246	1.2 (0.8, 2.1)	0.387
Married (ref = unmarried)	2.2 (2.9, 1.7)	<0.001	2.3 (1.6, 3.4)	<0.001
Mom drink during pregnancy (ref = no drinking)	0.8 (0.3, 1.6)	0.545	0.9 (0.4, 2.6)	0.827
Mom Smoke during pregnancy (ref = no smoking)	0.8 (0.6, 1.1)	0.236	1.2 (0.8, 1.9)	0.469
Maternal Race (ref = White)				
Black	3.2 (1.7, 6.0)	<0.001	3.2 (1.3, 8.7)	0.013
Native	0.3 (0.2, 0.4)	<0.001	0.3 (0.2, 0.4)	<0.001
Asian/PI	0.9 (0.5, 1.5)	0.602	0.7 (0.3, 1.4)	0.332
No Birth Defect on BC (ref = Birth defect)	0.7 (0.3, 1.4)	0.248	0.9 (0.4, 2.6)	0.889
Birth or pregnancy covered by Medicaid (ref = yes)	2.4 (1.8, 3.1)	<0.001	2.3 (1.7, 3.2)	<0.001
Father Name on BC (ref = no father listed)	0.8 (0.5, 1.3)	0.385	0.7 (0.3, 1.3)	0.209
Maternal age at birth (continuous)	1.0 (0.9, 1.0	0.281	1.0 (0.9, 1.0)	0.591
Maltreatment Report (ref = no maltreatment report)	0.6 (0.5, 0.9)	0.003	0.8 (0.5, 1.2)	0.275
Divorced/Separated 12 months before pregnancy (ref = no)	1.6 (1.1, 2.4)	0.018	1.3 (0.7, 2.1)	0.360
Reported moving 12 months before pregnancy (ref = no)	1.7 (1.3, 2.2)	<0.001	1.4 (1.0, 1.9)	0.048
Reported losing job 12 months before pregnancy (ref = no)	1.4 (0.9, 2.2)	0.111	1.9 (1.1, 3.2)	0.021
Reported partner/husband lost job 12 months before pregnancy (ref = no)	0.8 (0.5, 1.3)	0.426	1.0 (0.6, 1.8)	0.883

*OR* Odds Ratio

### Incidence proportion estimates and hazard ratios

**Fig. 3:**
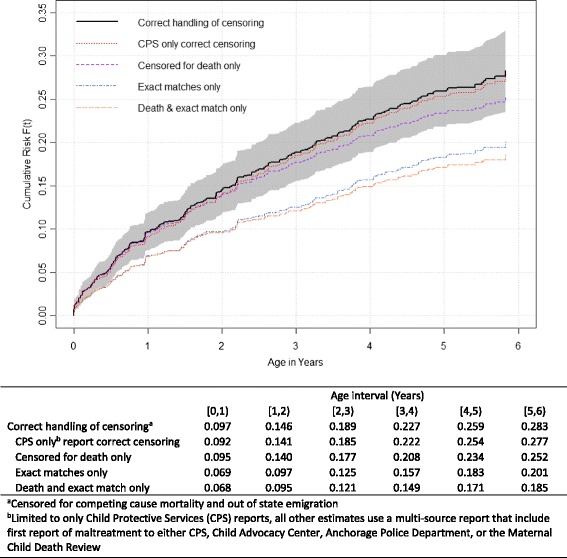
Risk of first maltreatment report, ALCANLink 2009 (*n* = 1235)

We observed that before the age of 6 years 28.3%_w_ (95% CI: 23.6%_w_, 33.0%_w_) of the 2009 births to Alaska residents were the subject of at least one multi-source maltreatment report. Under the non-linkage assumption for out-of-state emigration (assuming all non-linkages to any of the multi-source outcome agencies remained in the cohort outcome free) the incidence proportion calculated attenuated from 28.3%_w_ to 25.2%_w_, an absolute difference of 3.1%. When we restricted our analysis to deterministic linkages only, the incidence proportion calculated attenuated from 28.3%_w_ to 20.1%_w_, an absolute difference of 8.2%. Combining both sources of non-linkage error, the incidence proportion further attenuated to 18.5%_w_, an absolute difference of 9.8%. Finally, the incidence proportion calculated when restricted to using only child welfare reports (27.7%_w_; 95% CI: 23.0%_w_, 32.4%_w_) was nearly equivalent to the multi-source maltreatment report outcome **(**Fig. 3 **)**.

The hazard ratios for multiple risk and demographic factors were also influenced by failing to correctly account for censoring and/or restrictive data linkage (Table 4). Failing to account for out-of-state emigration underestimated the HR by 33% for military paid births (0.7 vs 1.1), and overestimated the HR by 11% for Alaska Native mothers (3.3 vs 3.0), and 10% for Medicaid births (4.1 vs 3.7). Limiting linkages to deterministic matches also resulted in biased HRs, with unmarried mothers (3.1 vs 3.8), and low maternal education (2.3 vs 3.1) all reporting underestimated HRs, and maternal smoking (3.6 vs 2.9) overestimating the HR. Combining both forms of error (failing to account for censoring and restrictive linkages), multiple factors and characteristics were both over and underestimated by 10% or more and include: military paid births, Alaska Native mothers, marital status, low education, child sex, young maternal age, maternal smoking during the 2 years before pregnancy, and reporting moving 12 months prior to birth.

**Table 4:** Comparing the hazard ratios of first maltreatment report before age six years accounting for out of state emigration and linkage parameters, ALCANLink (*n* = 1235)

Factor	Accounting for emigration (probabilistic)		Not accounting for emigration (probabilistic)		% change^a^	Accounting for emigration (deterministic)		% change^a^	Not accounting for emigration (deterministic)		% change^a^
	HR	95% CI	HR	95% CI		HR	95% CI		HR	95% CI	
Military paid births (ref = nonmilitary)	1.1	0.7, 1.8	0.7	0.4, 1.2	0.333	1.2	0.6, 2.1	0.081	0.7	0.4, 1.4	0.309
Race (ref = White)											
Alaska Native	3.0	2.2, 3.9	3.3	2.5, 4.4	0.114	3.0	2.2, 4.3	0.019	3.6	2.6, 5.0	0.204
Other	2.3	1.4, 3.8	2.3	1.4, 3.8	0.000	2.3	1.3, 4.3	0.006	2.4	1.3, 4.3	0.039
Unmarried marital status ref. = Married)	3.8	2.9, 5.1	4.1	3.1, 5.5	0.079	3.1	2.3, 4.4	0.177	3.1	2.2, 4.3	0.186
Medicaid birth (ref = no)	3.7	2.6, 5.2	4.1	2.9, 5.7	0.104	3.7	2.4, 5.6	0.005	4.0	2.6, 6.1	0.088
<12 years maternal education (ref = 12+ years)	3.1	2.3, 4.2	3.3	2.5, 4.5	0.058	2.3	1.6, 3.3	0.267	2.5	1.7, 3.6	0.212
Female (ref = Male)	1.3	1.0, 1.7	1.2	0.9, 1.6	0.024	1.2	0.9, 1.6	0.024	1.5	1.1, 2.0	0.152
Maternal age (ref = 25+ years)											
20–24 years	2.2	1.6, 3.0	2.2	1.6, 3.0	0.009	2.2	1.6, 3.0	0.009	2.2	1.6, 3.0	0.009
< 20 years	3.5	2.5, 5.1	3.7	2.5, 5.3	0.034	3.7	2.5, 5.3	0.034	3.1	2.1, 4.7	0.121
Birth weight < 2500 g (ref = 2500 + g)	1.7	1.4, 2.0	1.6	1.3, 1.9	0.060	1.7	1.3, 2.1	0.005	1.5	1.2, 1.9	0.096
Any Maternal smoking 2 years prior to birth (ref = No)	2.9	2.2, 3.9	3.0	2.2, 4.0	0.015	3.6	2.6, 5.0	0.225	3.5	2.5, 4.9	0.193
Any Birth defect listed on birth certificate (ref = No)	1.2	0.5, 2.8	1.2	0.5, 2.7	0.026	1.1	0.3, 3.3	0.125	1.1	0.4, 3.2	0.071
Mother Lost job^b^ (ref = No)	2.4	1.7, 3.5	2.4	1.6, 3.4	0.025	2.1	1.3, 3.4	0.124	2.3	1.5, 3.7	0.034
Moved to a new address^b^ (ref = No)	1.6	1.2, 2.1	1.6	1.2, 2.0	0.019	1.6	1.2, 2.2	0.012	1.4	1.0, 2.0	0.103

*HR* Hazard Ratio, *95% CI* 95% Confidence Interval, *ref* reference level

^a^% change compared to HR accounting for emigration and using probabilistic linkages

^b^During the 12 months before the child was born

## Discussion

We documented that failing to account for out-of-state emigration and/or using restrictive linkage methods in longitudinal birth linkage studies will bias both the incidence proportion and effect estimates. Integrating unique data resources in the state of Alaska enabled us to examine these sources of bias. The manageable sample size facilitated comprehensive high confidence data linkages and total cohort follow-up using the PFD. Furthermore, we demonstrated the utility of linking the PRAMS sampled child of a respondent mother with administrative data to effectively measure the incidence proportion of reported maltreatment over time in a representative birth population.

### Outcome ascertainment data sources

All administrative studies using official reports of maltreatment (reports to CPS) are affected by potential detection bias (Hussey et al. 2006; McGee et al. 1995). It is important to note that not all maltreatment occurring in this population is reported, and that not all reports are substantiated by child welfare. It is assumed that many cases of maltreatment are never reported for a wide variety of reasons, including failure to seek care, stigmatization, minimal contact with mandatory reporters, missed diagnosis, among other reasons (Gilbert et al. 2009; Delaronde et al. 2000; Gunn et al. 2005). We attempted to improve upon reliance on CPS records alone by including reports to Child Advocacy Centers, Anchorage Police Department, and Child Death Review records. However, in this sample, we found that CPS reports captured nearly all (98%) of the ascertained maltreatment reports, and these additional administrative sources had essentially no influence on incidence proportion estimates of any maltreatment.

Future linkage studies, when any reports are the outcome, may gain little utility by linking additional sources beyond CPS when all allegations of reports, regardless of screening determination and type are recorded and available through child welfare. However, it is clear that CPS records alone are an imperfect source of data for measuring child maltreatment and these conclusions may not apply to states with different types of child welfare agency structures (e.g. non-centralized) (Fallon et al. 2010). Other sources (not included in our study) may still be beneficial for increased detection of reports, for example medical records and self or proxy reported maltreatment obtained through survey (Robinson et al. 1997; Schnitzer et al. 2011; Turner et al. 2010; Finkelhor et al. 2009). A benefit of the ALCANLink methodology is that self/proxy reported maltreatment through survey can in theory be implemented through follow-up survey fairly easily. Alaska currently has a three-year follow-up survey to PRAMS and in 2016 (2013 PRAMS cohort) began asking questions about maltreatment experiences. Additional follow-up could also be done later in life for improved serial detection, and combined with administrative records would maximize ascertainment (Calderwood and Lessof 2009). Finally, because detection and reporting may be differential by maltreatment type additional research is needed to determine if maltreatment type produce the same patterns of bias as seen with any maltreatment, and if particular sources increase/decrease detection of specific maltreatment types.

In addition to increased detection, improving outcome ascertainment and classification is also needed. Consensus review by expert panels is a standardized process that could be used to improve the reliability and consistency of maltreatment classification (Schnitzer et al. 2004). Such panels are already used for child death review processes, and could be extended to non-fatalities and unlike full birth cohort studies are potentially feasible for PRAMS based maltreatment linkage studies that have a manageable sample size.

### Bias in incidence proportion

This study was able to achieve a high rate of follow-up through the first five years of life (especially for non-military paid births). Three quarters (75%) of the 2009 PRAMS births, representing 86% of the person-time of follow-up, had complete follow-up from birth to administrative censoring. High completeness of follow-up on the entire baseline population minimizes the potential for bias in estimating incidence proportion and effect estimates over time (Rothman 2012). Using the PFD and death data allowed us to investigate the assumption made in nearly all birth cohort linkages studies that subjects who do not link with CPS records remain in the cohort outcome free. As we detected an increasing bias with length of follow-up, longitudinal birth cohort linkage studies without an annual census equivalent to the PFD and with follow-up beyond 3-years may need to adjust their estimates by a scale factor to produce unbiased estimates. Clearly, out-of-state emigration likely varies from state to state which could lead to differences in the impact of the non-linkage assumption bias. One possible way to address this issue and estimate a scale factors would be to derive inverse-probability-of-censoring weights from the Alaska data. Although a state may have differential out-of-state emigration patterns, with a sufficiently large predictor set, the inverse probability of censorship weights from the Alaska data may be transferable and allow for improved accuracy in subgroup comparisons of the incidence proportion over time.

By limiting to the PRAMS population-based subsample (as opposed to the entire Alaskan 2009 birth cohort) we were able to set liberal manual review ranges and only automatically accept linkages with perfect matches on all linkage elements (first and last name, date of birth, sex, and residence). This resulted in high overall linkage success between sources with minimal effort, resources, and time. Studies linking entire birth cohorts may limit manual review and rely heavily on probabilistic cut points as a product of limited resources and data size resulting in unquantified sensitivity and specificity (Qayad and Zhang 2009). Variation in a state’s capacity and ability to integrate data could impact comparability of estimates produced through large scale data linkage projects. Deterministic linkages alone underestimate the incidence proportion of maltreatment, thus probabilistic methods are needed. Birth population studies that are unable to extensively manually review probabilistic linkages should consider quantifying the impact of mismatches within the probabilistic linkages, and adjust estimates accordingly. Furthermore, publishing full linkage methodology in supplemental material can allow other researchers to replicate methods and develop comparable estimates. The benefit of the ALCANLink methodology to conduct a longitudinal birth cohort linkage study is reflected in the manageable population representative and standardized PRAMS sample methodology utilized. These methods may be a viable option for states to consider and can be implemented in a largely systematic method allowing for improved comparability, regional, and even national assessments. Further development is needed to create a transferable platform for other PRAMS jurisdictions to utilize.

### Bias in hazard ratios

We confirmed that the hazard ratio will be biased for some estimates if out-of-state emigration is unaccounted for, or linkages are made overly restrictive (as in the extreme case of exact matches only). The direction and magnitude of the error associated with the bias depends on the three-way association between the exposure, outcome, and factor influencing linkage and therefore can produce estimates that over or underestimate the true effect. We detected that the bias associated with linkage method can be strong enough to “pull” the effect across the null (as in the case for military paid births). Because the direction and magnitude of the bias is not readily predictable, results produced without addressing these forms of bias could result in erroneous conclusions, especially when comparing subgroups.

### Comparison with prior published research

No national estimate is available for comparison of the incidence proportion estimate generated in the ALCANLink study and bias estimates measured. Researchers in California however, did observe that 14.8% of children born during 2006–2007 in the state were reported to child welfare before age 5 years (Putnam-Hornstein et al. 2014). They also reported that relative to White children, Native American children had 2.7 (95% CI: 2.6, 2.8) times the incidence proportion of being reported before age five (36.5% vs 13.7%). Using similar methodology to California’s estimates (not accounting for out-of-state emigration) we observed that 25.1%_w_ (95% CI: 21.0%_w_, 29.1%_w_) were reported to child welfare before age 5 years. This crude estimate is 1.7 times that of California. Similarly to California however, we observed that relative to White children, Alaska Native/American Indian (AN/AI) children had 2.8 (95% CI 2.3, 4.1) times the hazard of being reported before age five, with similar stratum specific estimates (41.3%_w_; 95% CI: 33.2% _w_, 49.3% _w_) for AN/AI children vs 15.8% _w_ (95% CI: 11.4% _w_, 20.2% _w_) for White children. Although Alaska indicated a crude elevated estimate relative to California, variations in population movement between these states could impact any direct comparison. Further, the observed similarity in the stratum specific estimates indicate confounding by race and that race standardization may be needed to account for large differences in underlying population distributions to facilitate state-by-state comparisons.

### Limitations

This study has a few notable limitations. 1) PRAMS respondents may be differential from the total sampled population resulting in selection bias. We conducted a post-hoc comparison with the full 2009 birth cohort and found a similar raw percentage of births reported to CPS suggesting a minimal impact on overall estimates. 2) This study accounted for censorship using the PFD based on a crude mid-year interval specification which may have led to erroneous or imprecise exclusions which could result in an overestimation of out-of-state emigration. The impact of this on our person-time estimation is unknown but could in theory result in an overestimation. However, we feel that the overall impact is likely minimal as we conducted extensive data mining from all available systems for respondents that failed to match with the PFD. Further, for those that had “breaks in PFD applications” for example applied in 2010 and again in 2011 but not in 2009 we assumed they remained in the state even though we were able to document for some cases intermittent movement (e.g. attendance at out-of-state school). Thus our conservative censoring rule may in fact still overestimate actual eligible person-time in the state for the population and would likely lead to attenuated results. 3) The multi-agency outcome measure was limited due to incomplete law enforcement and CAC data.

## Conclusion

Child maltreatment is a substantial public health problem; however, etiologic analyses are needed to inform public health prevention efforts. Comprehensive population-representative data linkage studies are essential to detangling the multifaceted etiologies and interplay of factors that contribute to child maltreatment. Further, our confidence in assessing the impact of public health prevention efforts and policy over time relies on reliable, consistent estimates. PRAMS provide a rich set of measures for prospective cohort studies and when linked with administrative sources (such as Medicaid claims, hospital visits, and follow-up surveys) can efficiently increase the breadth of information available for longitudinal analysis. Other PRAMS states should consider the utility of the ALCANLink methodology for studying reported child maltreatment longitudinally. This study underscores the importance of manual review of data linkages to monitor linkage quality and suggests the need for increased transparency and standardizations in linkage studies. We also highlight the importance of adjustment for out-of-state emigration, especially for states like Alaska that may have large population movements among population subsets. Data linkage did not substantially improve the detection of reported maltreatment in this study; additional research is needed to develop methods to improve the identification and classification of maltreatment.

## Additional file

Additional file 1: Detailed information regarding cohort follow-up and censoring, maltreatment classification, and data linkage methods. (DOCX 161 kb)

## Abbreviations

ALCANLink: Alaska longitudinal child abuse and neglect linkage project
CAC: Child advocacy center
CDC: Centers for disease control and prevention
CDR: Child death review
CPS: Child protective services
HR: Hazard ratio
PFD: Permanent fund dividend
PRAMS: Pregnancy risk assessment monitoring system
SCAN: Surveillance of child abuse and neglect
US: United States
