# Supplementary material for: Quantifying sources of bias in longitudinal data linkage studies of child abuse and neglect: measuring impact of outcome specification, linkage error, and partial cohort follow-up
Source: Inj Epidemiol. 2017 Aug 7;4:23. doi: 10.1186/s40621-017-0119-6 (PMC5545181; doi:10.1186/s40621-017-0119-6)
Supplement: Additional file 1: — Detailed information regarding cohort follow-up and censoring, maltreatment classification, and data linkage methods. (DOCX 161 kb) [file 40621_2017_119_MOESM1_ESM.docx]

**Supplemental Material**

The process of developing the Alaska Longitudinal Child Abuse and Neglect Linkage (ALCANLink) project resulted in multiple validation and subsidiary analyses to quantify assumptions or other decisions being made. This supplemental material provides in-depth details regarding some of the key points referenced in the main manuscript allowing for a more in-depth scrutiny or replication of our methods.

**Cohort Follow-up and Censoring**

**Figure S1** depicts the censoring rules developed. We examined multiple options for censoring rules to determine the impact of different ways of defining censored on 1) the person-time estimation, 2) number of outcomes (reports of maltreatment) excluded based on the rule specification (recognizing that such exclusions may reflect lack of precision in the PFD and CPS dates), and 3) the impact on the incidence estimate, crudely approximated as the number of events divided by the total person-time at risk **Table S1.** Based on the rules selected, we captured all outcomes within our observation window and censored for out-of-state emigration using a conservative rule to maximize accrual of person-time.

Subjects censored due to competing causes of death (deaths not classified as maltreatment-related by the MCDR committee) were followed until the date of death. We assumed all PRAMS respondents were present in Alaska thru 12/31/2009 unless a) no match with any PFD was made for any subsequent year (n=59) or b) matches were made with PFD years but only with out-of-state addresses (n=14). These 73 exceptions were censored after 3 months from the date of birth. Finally, we used the last PFD year that matched with an Alaskan address as the censoring year and assumed continual residence in Alaska until this event. If the last PFD linkage in Alaska was during 2011, 2012, 2013, or 2014 PFD years we censored at the mid-year of the subsequent calendar year after the last documented Alaska residence assuming emigration follows a uniform distribution. We reasoned that (for example) a 2011 PFD year application reflects residence during the 2010 calendar year, to be eligible for the 2010 PFD the subject must have physically resided in Alaska (aside for a few exceptions) the majority of that year and lost eligibility sometime during the subsequent year (2011). Finally, we administratively ended follow-up for the study at 12/31/2014 (n=908) for all those remaining in the cohort as evidenced by a linkage with the 2015 PFD.

**Figure S1.** ALCANLink follow-up and person-time specification profiles

**Table S1.** Ascertainment of the 327 identified multi-source maltreatment reports observed during follow-up and impact of censoring rule on incidence per 100 person-years, ALCANLink 2009 (n=1235)

| **Approach** | **Outcomes excluded** | **Estimated person-time** | **Estimated Rate per 100 person-years (95%CI)^a^** | |
| --- | --- | --- | --- | --- |
| Censoring only deaths | 0 | 5521.8 | 5.9 (5.3, 6.6) | |
| Censor deaths and all nonAK specified or missing PFD years | 32 | 4455.7 | 6.6 (5.9, 7.4) | |
| Censor deaths and all nonAK specified or missing PFD years after last AK PFD year | 6 | 4654.8 | 6.9 (6.1, 7.6) | |
| Censor deaths and all nonAK specified or missing PFD years after last AK PFD year, allow 3months for loss during first year, and 6 months for nonlinkage loss in subsequent years^b^ | 0 | 4785.4 | 6.8 (6.1, 7.6) | |
| ^a^Unweighted estimates  ^b^Method selected for this analysis | | | |  |

**Maltreatment classification**

Documenting and classifying child maltreatment is a complicated process.(1-6) Research suggests that official determinations by child welfare underestimate the true magnitude of occurrence in a population.(2) When only administrative source information is available some research indicates that using reports/allegations regardless of screening and in combination with other source information may increase the identification of children who may experience some form of child harm or victimization.(3) We choose to use any reported maltreatment from all reporting agencies (child protection, law enforcement, child advocacy center, and child death review) in effort to improve our detection of children who may have experienced child maltreatment. It is clear that this definition will include false positive and false negative cases, however research limited to child welfare reports does suggest that children and families who are reported.(4-6) Under this reasoning, the reported maltreatment serves as a proxy measure of children who are likely to have some degree of maltreatment. **Table S2** presents our classification determinations from each of the agencies records, and our decisions for how we classified cases.

**Table S2.** Agency maltreatment report classification determinations

| **Code** | **Agency** | **Description** | **Report Classification** | **Report exclusions** |
| --- | --- | --- | --- | --- |
| CPS | Office of Children’s Services | Single centralized statewide agency and data system. | Presenting alleged maltreatment as classified by OCS physical abuse, sexual abuse, mental injury and neglect. Includes both screened in and out reports | Multiple reports on same incident and referrals out of state |
| APD | Law Enforcement | Only includes the Anchorage Police Department | Reports of victims with a nature of call indicated as abuse, assault, homicide, or child abuse were classified as physical abuse. Reports of rape, sexual abuse of a minor or other sexual exposure (attempted or otherwise) or offensive touching were classified as sexual abuse. Exposure to domestic violence and threatening were classified as mental injury. Neglect, DUI, reckless endangerment, Kidnap, and undetermined and negligent homicide deaths were classified as Neglect. | Reports with child listed as perpetrator, informational reports if occurring in isolation, medical assistance, and all reports unrelated to physical or sexual abuse, neglect, and mental injury |
| CAC | Child Advocacy Center | Includes 8 of the 10 active CAC's in the State of Alaska affiliated with the Alaska Children’s Alliance | Presenting alleged maltreatment as classified by the CAC as physical abuse, sexual abuse and neglect. Drug endangered includes as neglect, and witness to violence classified as mental injury. | Only extract cases considered alleged victims by the CAC |
| MCDR | Maternal Child Death Review | Single centralized statewide review and data system. | Consensus review committee classifies the death was "yes" or "yes probably" caused or contributed to by abuse, neglect, or negligence. | Unknown but committee suspicious classification not included as a case |

**Data linkages**

We performed direct and probabilistic record linkages between PRAMS and each datasets. Prior to all data linkages we conducted minimal systematic record cleaning on each dataset, which included 1) equalizing dates of birth to the same length and short date format of YYYYMMDD, 2) removing all special characters, punctuation, and leading or trailing spaces from linkage variables, 3) remove all missing character values such as 99, “NULL”, or “Missing” from all strings, 4) Removing prefixes and suffixes, and 5) standardizing all text case.(7)

We conducted iterative linkage processing which reduces the amount of suspected matches by removing confirmed matches from probability match scoring. Deterministic linkages require field values from both record sets to be completely identical and in the same format (e.g. string or numeric) for a positive match. Deterministic linkages can be conceptualized as a Boolean distance of either 0 (False) or 1 (True). Due to this restriction, direct linkages have excellent specificity but reduced sensitivity due to potential false negative matches due to typos, transpositions, and deletions.(8)

Each PRAMS respondent was direct linked to an Alaska birth record by a birth certificate number and CUBS survey by a unique research ID shared with PRAMS. All subsequent probabilistic linkages were based on distance metrics and used date of birth, infant last and first name, alias names, and sex. Due to the relatively small population size of Alaska, few identifiers are needed to make reliable probabilistic linkages. We created a master name list in long format with all known name variations of each subject to reduce manual review and improve linkage efficiency. The probability of an erroneous exact match however, increases with the number of alias names included. To guard against this we manually reviewed all linkages where the birth names, date of birth and sex as indicated on the birth certificate were not exact (i.e we compared the deterministic linkage with no aliases with confirmed probabilistic linkages). Finally, we used a de-duplication process (which links each record in a record set against all other records) to identify potential mismatches based on names, date of birth and sex. We manually reviewed and correctly specified any mismatches where records shared the same identifiers.

Using the RecordLinkage package(9) in R (10) we built comparison patterns based on a Jarowinkler distance metric.(11) We then created probability match scores that weight the probability of the distance score based on the average frequency and error rate of the pairs.(12-14)

To determine manual review thresholds we conducted an initial linkage calibration exercise by sampling 100 subjects form the PRAMS sample and creating a duplicate dataset with known linkage miss-specifications. We used this training dataset to develop initial probability match scores to establish a classification interval based on the generalized Pareto distribution, by identifying the “long tail” of the distribution. The Pareto distribution belongs to a family of continuous density distributions that bind the “heavy or fat-tailed” distribution to specified distribution tails and useful for data linkage where the majority of matches have low probability match scores.(15). Visually, the interval of 0.75 – 0.95 was indicated for manual review, with 0.82 suggested as the lower threshold from the distribution (See **Figure S2** for the mean residual life plot which plots the mean residual estimated from the Pareto distribution against the probability match score). We manually reviewed all records with a match score above 0.75 to validate the suggested threshold. From this exercise we detected that the probability of a false negative match below 0.82 is <0.1%.

**Figure S2.** Mean Residual Life plot, of the linkage probability used to visually identify the “long-tail” of the distribution to establish initial manual review thresholds for manual review of data linkages


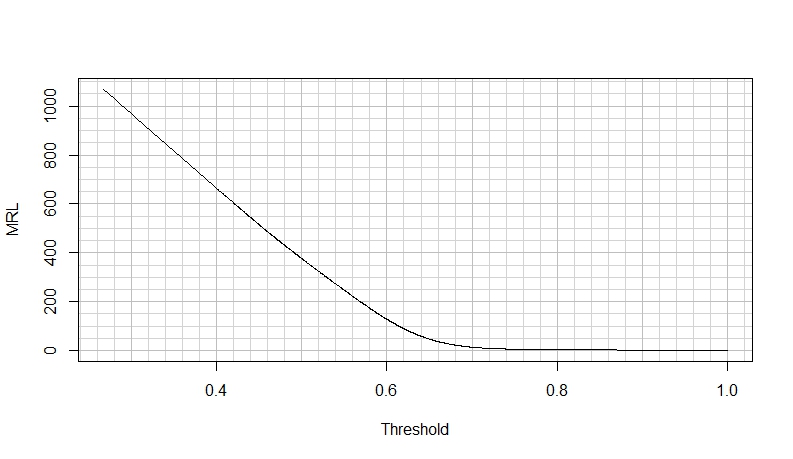


For each data linkage with the PRAMS cohort we calculated the suggested lower threshold from the generalized Pareto distribution and manually reviewed all probability match scores between this value and 0.99. All suggested matches within the probability match score interval were manually reviewed and classified as linkages or nonlinkages according to standardized criteria. We calculated the linkage rates for each source (if applicable) by calculating the proportion of successful matches with linkage rates and manual review ranges specified (see **Table S3** below). In essences the goal of this exercise was to use probability linkage methods to identify a reasonable lower bound threshold for manual reviews, as we only automatically accepted exact matches opposed to establishing a probabilistic acceptance limits where some score <100 will be accepted automatically.

*CPS linkage comparison between PRAMS sample and statewide birth population*

We compared the proportion of subjects with a child protective services report between the PRAMS sample and full birth cohort. To limit the amount of data linkages within the full cohort we used the end point of first reported allegation of harm to the States child protective services agency. We linked 11,033 PRAMS eligible births in 2009 to CPS reports and estimated the prevalence of births that had at least one CPS report of harm during the study window.

We calculated the expected false rejection and false acceptance by simulating 10 draws of size 1,000 from a training dataset with the following parameters: rejection region specified as < 0.95, and automatic acceptance region 0.95 and greater. From the simulation exercise we estimated the proportion of expected false positive matches within the automatic acceptance region (0.122) and proportion of expected false negative matches in the automatic rejection region (0.018). We then applied these estimates to adjust the linked data numbers to estimate the expected proportion of all 2009 births that link to CPS during the study period.

Among the 11,033 PRAMS eligible births, 2,533 linked to CPS with a first report by 12/31/2014. After accounting for simulated weights of expected false acceptance and false rejection in specified regions, the estimated number of positive CPS linkages in the birth cohort = (2533 - 379*0.122 + 2474*0.018) = 2,531. An estimated 23% of the PRAMS eligible births (n=11,033) linked to CPS compared to 24%_w_ of the PRAMS cohort. Thus the PRAMS sample is an adequate representation of the birth population and reflects an equal proportion of subjects reported to CPS. This is important as nonresponse could potentially be differentially associated with experiencing the outcome under study and thus result in biased estimates.

**Table S3.** Linkage rates by data source, ALCANLink 2009 (n=1,235)

| **Source** | **Records^a^** | **Review threshold^b^** | **Exact match** | **Records Reviewed** | **Confirmed**  **n (%)^c^** | **Total Linkage matches** | **Proportion of PRAMS sample** | **Proportion of records^d^** |
| --- | --- | --- | --- | --- | --- | --- | --- | --- |
| **Core cohort linkages** | | | | | | | | |
| PFD 2010 | 10298 | 0.85 - 0.99 | 1036 | 417 | 54 (12.9) | 1090 | 0.883 | 0.106 |
| PFD 2011 | 10716 | 0.89 - 0.99 | 1055 | 278 | 63 (22.7) | 1118 | 0.905 | 0.104 |
| PFD 2012 | 10650 | 0.89 - 0.99 | 999 | 235 | 58 (24.7) | 1057 | 0.856 | 0.099 |
| PFD 2013 | 10428 | 0.89 - 0.99 | 941 | 223 | 61 (27.4) | 1002 | 0.811 | 0.096 |
| PFD 2014 | 10456 | 0.89 - 0.99 | 915 | 201 | 64 (31.8) | 979 | 0.793 | 0.094 |
| PFD 2015 | 10362 | 0.85 - 0.99 | 888 | 205 | 48 (23.4) | 936 | 0.758 | 0.090 |
| CPS link | 3203 | 0.84 - 0.99 | 262 | 406 | 57 (14.0) | 319 | 0.258 | 0.100 |
| CAC link | 395 | 0.89 - 0.99 | 29 | 93 | 14 (15.1) | 43 | 0.035 | 0.109 |
| APD link | 375 | 0.89 - 0.99 | 17 | 120 | 16 (13.3) | 34 | 0.028 | 0.091 |
| Vital Death Records | 68 | direct match | 68 |  |  | 23 | 0.019 | 0.338 |
| **Additional completed and provisional source linkages** | | | | | | | | |
| **Child** | | | | | | | | |
| Medicaid | 8443 | 0.85 - 0.99 | 662 | 215 | 58 (27.0) | 720 | 0.583 | 0.085 |
| AKAIMS | 334 | Provisional^e^ |  |  |  | 7 | 0.006 | 0.021 |
| DSDS | 195 | Provisional |  |  |  | 28 | 0.023 | 0.144 |
| **Mother** |  |  |  |  |  |  |  |  |
| CPS victim | 52977 | 0.89 - 0.99 | 204 | 42 | 25 | 229 | 0.185 | 0.004 |
| CPS any |  | Provisional |  |  |  | 541 | 0.438 | 0.009 |
| APD | 5061 | 0.89 - 0.99 | 38 | 48 | 6 | 44 | 0.036 |  |
| Medicaid |  | Provisional |  |  |  | 840 | 0.680 |  |
| DJJ |  | Provisional |  |  |  | 230 | 0.186 |  |
| AKAIMS |  | Provisional |  |  |  |  | 0.084 |  |
| DSDS |  | Provisional |  |  |  |  | 0.013 |  |
| **Father** |  |  |  |  |  |  |  |  |
| CPS victim | 48035 | 0.89 - 0.99 | 103 | 143 | 17 | 120 | 0.097 | 0.002 |
| CPS any |  | Provisional |  |  |  | 351 | 0.284 | 0.005 |
| APD | 6279 | 0.89 - 0.99 | 27 | 49 | 2 | 29 | 0.023 |  |
| Medicaid |  | Provisional |  |  |  | 587 | 0.475 |  |
| DJJ |  | Provisional |  |  |  | 201 | 0.163 |  |
| AKAIMS |  | Provisional |  |  |  | 51 | 0.041 |  |
| DSDS |  | Provisional |  |  |  | 8 | 0.007 |  |
| NOTE: AKAIMS = Alaska’s Automated Information Management System that captures statewide behavioral health data; CPS = Child Protective Services; APD = Anchorage Police Department; DJJ = Division of Juvenile Justice; DSDS = Division of Senior and Disability Services  **^a^**The number of unique individual records in the specified source with a birth year of 2009  ^b^Review threshold is the calculated probability match score. The base probability match score was established through machine learning iterative processing to establish where the true match probability  ^c^Number of confirmed linkage matches based on manual reviews of suspected linkages within the review threshold between records, proportion of reviewed records confirmed.  ^d^Proportion of the PRAMS sample that matches to the record source.  ^e^Provisional matches were based on a manual look-up of all records in a centralized client index within the Alaska Division of Health and Social Services. This system only included presence or absence within data systems, did not allow for batch probabilistic linkage and was based on a static instance of these data. | | | | | | | | |

(1) Socolar RR, Runyan DK, Amaya-Jackson L. Methodological and ethical issues related to studying child maltreatment. J Fam Issues 1995;16(5):565-586.

(2) US Department of Health and Human Services, Administration for Children and Families, Administration on Children, Youth and Families, Children's Bureau. Child Maltreatment 2013. 2015.

(3) Putnam-Hornstein E, Needell B. Predictors of child protective service contact between birth and age five: An examination of California's 2002 birth cohort. Children and Youth Services Review 2011;33(8):1337-1344.

(4) Dubowitz H, Pitts SC, Litrownik AJ, Cox CE, Runyan D, Black MM. Defining child neglect based on child protective services data. Child Abuse Negl 2005 May;29(5):493-511.

(5) Runyan DK, Cox CE, Dubowitz H, Newton RR, Upadhyaya M, Kotch JB, et al. Describing maltreatment: do child protective service reports and research definitions agree? Child Abuse Negl 2005;29(5):461-477.

(6) Hussey JM, Marshall JM, English DJ, Knight ED, Lau AS, Dubowitz H, et al. Defining maltreatment according to substantiation: distinction without a difference? Child Abuse Negl 2005;29(5):479-492.

(7) Randall SM, Ferrante AM, Boyd JH, Semmens JB. The effect of data cleaning on record linkage quality. BMC Med Inform Decis Mak 2013 Jun 5;13:64-6947-13-64.

(8) Tromp M, Ravelli AC, Bonsel GJ, Hasman A, Reitsma JB. Results from simulated data sets: probabilistic record linkage outperforms deterministic record linkage. J Clin Epidemiol 2011;64(5):565-572.

(9) Sariyar M, Borg A. The RecordLinkage package: Detecting errors in data. The R Journal 2010;2(2):61-67.

(10) R Core Team. R: A Language and environment for statistical computing. 2014;3.1.0.

(11) Jaro MA. Probabilistic linkage of large public health data files. Stat Med 1995;14(5‐7):491-498.

(12) Contiero P, Tittarelli A, Tagliabue G, Maghini A, Fabiano S, Crosignani P, et al. The EpiLink Record Linkage Software Presentation and Results of Linkage Test on Cancer Registry Files. Methods Inf Med 2005;44(1):66-71.

(13) Dunn HL. Record Linkage. Am J Public Health Nations Health 1946 Dec;36(12):1412-1416.

(14) Gu L, Baxter R, Vickers D, Rainsford C. Record linkage: Current practice and future directions. CSIRO Mathematical and Information Sciences Technical Report 2003;3:83.

(15) Arnold BC. Pareto distribution. : Wiley Online Library; 1985.
